# Pooling sputum testing to diagnose tuberculosis using xpert MTB/RIF and xpert ultra: a cost-effectiveness analysis

**DOI:** 10.1186/s12879-023-08330-9

**Published:** 2023-05-22

**Authors:** Vibol Iem, John S. Bimba, Victor S. Santos, Jose Dominguez, Jacob Creswell, Silaphet Somphavong, Tom Wingfield, Jahangir A. M. Khan, Luis E. Cuevas

**Affiliations:** 1 National Tuberculosis Control Center, Lao People’s Democratic Republic, Vientiane, Laos; 2 Liverpool School of Tropical Medicine, Pembroke Place, Liverpool, L3 5QA UK; 3 Zankli Research Centre, Bingham University, Karu, Nigeria; 4 Departamento de Medicina, Universidade Federal de Sergipe, Lagarto, Brazil; 5 Programa de Pós-Graduação em Ciências da Saúde, Universidade Federal de Sergipe, Aracaju, Brazil; 6 Institut d’Investigació Germans Trias i Pujol, CIBER Enfermedades Respiratorias, Universitat Autònoma de Barcelona, Barcelona, Spain; 7 Stop TB Partnership, Innovations and Grants, Geneva, Switzerland; 8 Lao Association for Medical Laboratory Sciences, Lao People’s Democratic Republic, Vientiane, Laos; 9 WHO Collaborating Centre for Tuberculosis and Social Medicine, Department of Global Public Health, Karolinska Institutet, Solna, 17177 Sweden; 10 Health Economics and Policy Unit, School of Public Health and Community Medicine and Centre for Health Governance, University of Gothenburg, Gothenburg, Sweden

**Keywords:** Tuberculosis, Xpert MTB/RIF, Xpert Ultra, Pooling, Cost-effectiveness, Cost-minimization analysis

## Abstract

**Background:**

The World Health Organization (WHO) recommends the diagnosis of tuberculosis (TB) using molecular tests, such as Xpert MTB/RIF (MTB/RIF) or Xpert Ultra (Ultra). These tests are expensive and resource-consuming, and cost-effective approaches are needed for greater coverage.

**Methods:**

We evaluated the cost-effectiveness of pooling sputum samples for TB testing by using a fixed amount of 1,000 MTB/RIF or Ultra cartridges. We used the number of people with TB detected as the indicator for cost-effectiveness. Cost-minimization analysis was conducted from the healthcare system perspective and included the costs to the healthcare system using pooled and individual testing.

**Results:**

There was no significant difference in the overall performance of the pooled testing using MTB/RIF or Ultra (sensitivity, 93.9% vs. 97.6%, specificity 98% vs. 97%, p-value > 0.1 for both). The mean unit cost across all studies to test one person was 34.10 international dollars for the individual testing and 21.95 international dollars for the pooled testing, resulting in a savings of 12.15 international dollars per test performed (35.6% decrease). The mean unit cost per bacteriologically confirmed TB case was 249.64 international dollars for the individual testing and 162.44 international dollars for the pooled testing (34.9% decrease). Cost-minimization analysis indicates savings are directly associated with the proportion of samples that are positive. If the TB prevalence is ≥ 30%, pooled testing is not cost-effective.

**Conclusion:**

Pooled sputum testing can be a cost-effective strategy for diagnosis of TB, resulting in significant resource savings. This approach could increase testing capacity and affordability in resource-limited settings and support increased testing towards achievement of WHO End TB strategy.

## Introduction

Tuberculosis (TB) was the second leading cause of death by an infectious disease after Coronavirus disease 2019 (COVID-19) [1]. The World Health Organization (WHO) recommends to provide upfront molecular tests (mWRDs) for the diagnosis of TB and at least rifampicin resistance to all individuals with presumptive TB [2]. mWRDs include the Xpert MTB/RIF [3] (MTB/RIF) and Xpert MTB/RIF Ultra (Ultra) [4], which are semi-automated and simultaneously detect *Mycobacterium tuberculosis* complex and markers of rifampicin resistance using the GeneXpert platform. The Ultra assay is currently the recommended Xpert assay, based on its increased sensitivity, which improves the detection of paucibacillary TB [5]. Several high TB burden countries such as South Africa and Uganda have transitioned towards use of Xpert as the upfront test for TB diagnosis. However, despite efforts made by National TB Programmes, mWRDs are still not used globally as the upfront test for TB diagnosis for many people. This is because of the high cost ($US 9.98 per test at FIND negotiated price) and mWRDs being predominantly available only at higher levels of the TB laboratory network with better infrastructure and more qualified human resources [6]. Consequently, due to the high costs of the test, cartridges are often rationed, and many tests are only used as reflex tests once people have been diagnosed, and more centralized testing can lead to longer turnaround time.

**Fig. 1:**
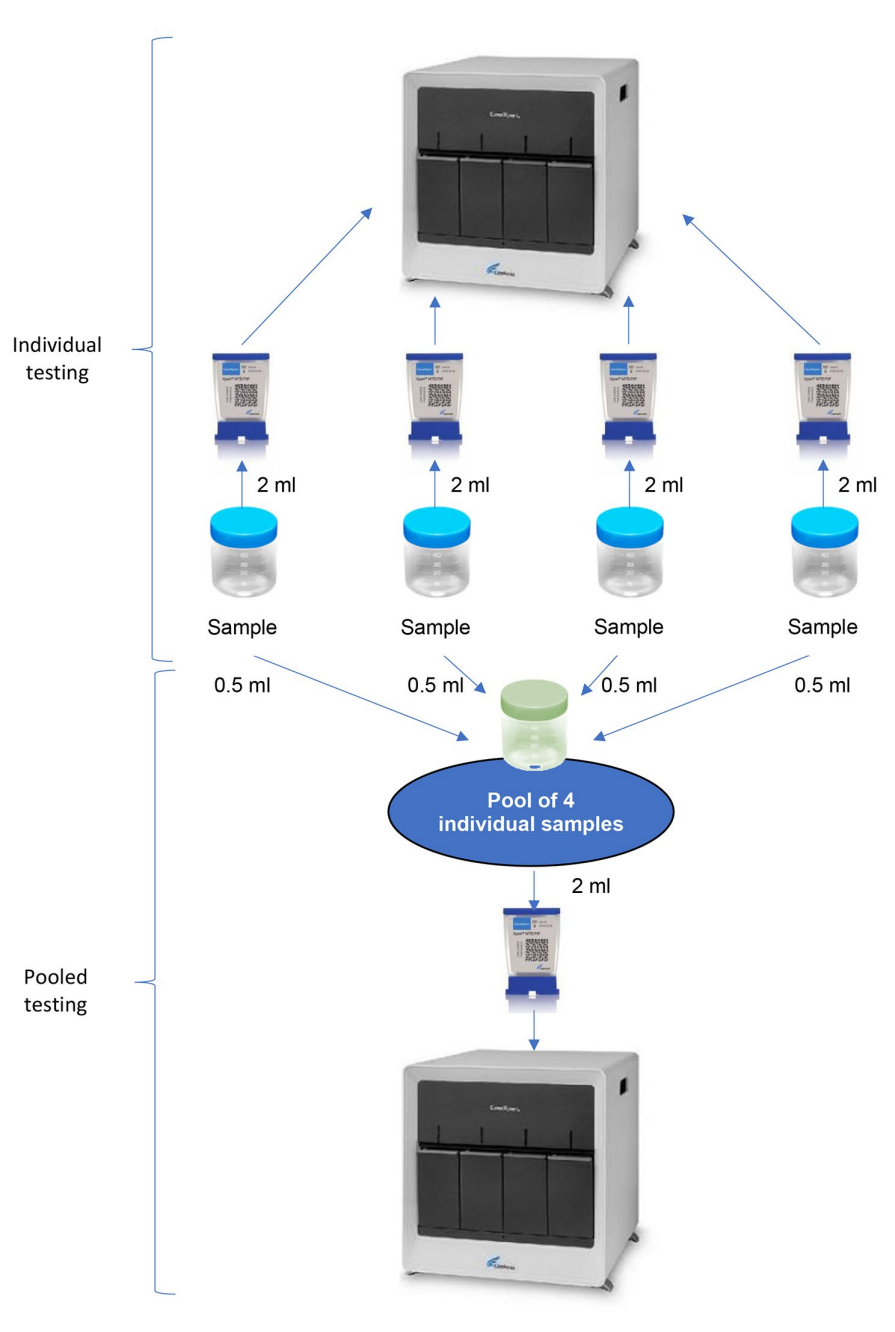
Flow diagram of the sputum processing* *Same test was used for individual and pooled testing (either Xpert MTB/RIF or Xpert Ultra).

To maintain sufficient TB testing capacity and cope with these challenges, one practice that has re-emerged during the Covid-19 pandemic is pooled testing. In this approach, several specimens collected from different presumptive TB cases are pooled (mixed) together and tested as a group in a single assay. If the pooled test is negative, it is then assumed all samples included in the pool are negative. If the pooled test is positive, it means at least one sample included in the pool is positive, and individual re-testing of samples is needed to identify the positive sample(s) (Fig. 1). A systematic review published in 2021 concluded this method was highly sensitive and specific and can substantially increase testing capacity with savings up to 27–31% in cartridges alone, depending on the prevalence of TB in the population tested [7]. However, data on cost-effectiveness are currently limited to assay savings on the basis of the number of cartridges that would have been required to test all specimens when using individual vs. pooled testing as part of individual evaluations.

In this study, we conducted a cost-effectiveness analysis of the pooled testing strategy of Xpert MTB/RIF in comparison with Xpert Ultra, during passive case finding (PCF) routine activities. Between each method, we compared the costs to test 1,000 patients, the potential resources savings, the diagnostic accuracy, the cost to detect one person with bacteriologically confirmed TB, and the potential increase in testing capacity and TB case detection.

## Materials and methods

In this cost-effectiveness analysis, a total of 3,076 individuals with presumptive TB were enrolled from two studies conducted in Lao PDR (840 individuals per study) [8], two studies in Nigeria (500 individuals per study) (Bimba et al. in press), and one study in Brazil (396 individuals) [9], which are described in more detail below.

WHO defines an individual with presumptive TB as anyone who shows symptoms or signs suggestive of TB. The most common symptom of pulmonary TB is persistent, productive cough, often accompanied by other non-specific respiratory symptoms (shortness of breath, chest and back pains, hemoptysis) and/or constitutional symptoms (loss of appetite, weight loss, fever, night sweats, and fatigue) however screening tests such as chest x-ray can also be used to identify people with presumptive TB despite lack of symptoms [10].

Pools were created by mixing four consecutive samples. Pooled samples were then tested with Xpert MTB/RIF or Ultra assays. Pools and their corresponding individual results were compared to determine the level of agreement.

Studies were cross-sectional surveys, conducted during PCF programmatic activities. In this approach, which is a patient-initiated pathway to TB diagnosis, individuals with symptoms suggestive of TB present spontaneously to the health facility for the health worker to initiate the investigation for TB using a diagnostic algorithm with sufficient sensitivity and specificity to diagnose TB [11].

In Lao PDR [8] and Nigeria (Bimba et al. in press), two independent studies were conducted during PCF of people with presumptive TB, one using Xpert MTB/RIF, and the other Ultra. The study from Brazil assessed the performance of pooled testing with Xpert Ultra only [9].

### Statistical analysis

Categorical data were summarized using descriptive statistics and chi-squared tests were used to test for statistically significant differences, where appropriate. Pooled test results (MTB-positive or MTB-negative) were compared with the four corresponding Xpert MTB/RIF or Xpert Ultra individual test results and their agreement was assessed by calculating the Kappa coefficient. The kappa values and their interpretations were as follows: <0, no agreement; 0–0.19, very weak agreement; 0.20–0.39, weak agreement; 0.40–0.59, moderate agreement; 0.60–0.79, substantial agreement; and 0.8–1.0, excellent agreement [12].

### Cost-effectiveness analysis

We measured the cost-effectiveness of pooled testing vs. individual testing by comparing the number of individuals that would be bacteriologically confirmed using each method.

Cost analysis is a technique that involves the systematic collection, categorization, and analysis of costs of any intervention [13]. Potential savings were calculated by comparing all resources required to test all specimens using pooled and individual testing by analyzing the costs of each TB detection method. We used an ingredient-based, top-down approach, in which all categories of inputs were listed alongside all quantities needed to perform all tests annually, for both the individual and pooled testing approach (Table 1).

The GeneXpert instruments set, biosafety cabinet and autoclaves, and other small equipment (uninterruptible power supply, timer, vortex) were considered as “capital items”. The cost of equipment was determined by using the estimated lifetime of capital items in years to which we then applied an annuity factor to estimate the cost per year. The useful time of the capital items reported here was based on annual warranty cost with a 5-year expected lifetime [14].

We also listed and quantified all recurrent items needed to perform all the tests over one year, with the cost of all items needed annually. The base level cost of MTB/RIF and Ultra testing were the same. All the Xpert cartridges, laboratory supplies, disposable personal protective equipment, biosafety supplies, and human resources were considered as “recurrent items”. We then divided the total annual cost for capital and recurrent items by the number of tests performed annually to estimate the unit cost to perform one test. Values for each country were adjusted for international dollars by using DEC (World Bank’s Development Economics department) alternative conversion factor (local currency units per US$) and purchasing power parity conversion factor, gross domestic product (local currency units per international $) from the World Bank (2021 data).

We then compared both approaches to calculate the difference in the money invested for testing 1,000 consecutive individuals, the number of people who could be tested for TB when using a fixed amount of 1,000 cartridges, and the costs per bacteriologically confirmed TB case detected. The cost of pooled testing also included the cost of retesting all specimens from positive pools individually. Thus, our cost-effectiveness analysis was able to demonstrate a cost-saving outcome if pooled testing cost less than individual testing while detecting at least the same or higher numbers of TB cases.

**Table 1:** Resources costs assumptions for TB diagnosis by Xpert MTB/RIF and Ultra (unit costs from the Global Drug Facility products catalogs “Ordering List of TB Medicines or Diagnostics, Medical Devices and other health products”)

	Price (US$)	Cost per test (US$)	Cost per test (International $)		
			Lao PDR	Nigeria	Brazil
**Supplies required for Xpert test: Provided by Cepheid**					
GeneXpert instrument	15,105	0.5815	1.977	1.522	1.242
Laptop HP or DELL brand	2395	0.1126	0.383	0.295	0.240
**Biosafety equipment**					
Laboratory coats	20	0.0018	0.006	0.005	0.004
Autoclave	22,000	0.5042	1.714	1.320	1.077
**Equipment**					
UPS 1500 VA	1,500	0.0577	0.196	0.151	0.123
Timer	30	0.0012	0.004	0.003	0.002
Vortex	220	0.0085	0.029	0.022	0.018
**Stationery**					
Indelible labelling marker	2	0.0033	0.011	0.009	0.007
Pens (red and blue or black)	1	0.0017	0.006	0.004	0.004
**Supplies required for Xpert test**					
MTB/RIF cartridges	499	9.9800	33.930	26.130	21.312
**Laboratory supplies**					
Sterile screw-capped specimen collection containers	83.5	0.0835	0.284	0.219	0.178
Paper towels	2	0.0067	0.023	0.017	0.014
**Personal protective equipment**					
Disposable gloves	20	0.0800	0.272	0.209	0.171
Surgical masks	21.5	0.0344	0.117	0.090	0.073
**Biosafety supplies**					
Disposable autoclave bags (LxW = 35"x25”)	20	0.0320	0.109	0.084	0.068
Disposable autoclave bags (LxW = 19"x14”)	20	0.0160	0.054	0.042	0.034
Tuberculocidal disinfectant solution 0.003 L per test	45	0.0270	0.092	0.071	0.058
**Human resources**					
Laboratory technician (40 h/week, 4 weeks/month)*	250	0.5000	1.700	1.309	1.068
**Total number of test/years**	6000*	**11.7141**	**40.91**	**31.50**	**25.69**
* Lao PDR data					

## Results

### Pooled testing diagnostic accuracy (Table 2)

In Lao PDR, in the Xpert MTB/RIF survey, 77/81 (sensitivity 95.1%, 95%CI 87.8-98.6%) pools containing ≥ 1 positive sample tested MTB-positive and 4/81 (4.9%, 95%CI 1.4-12.2%) tested MTB-negative. All 129/129 pools containing MTB-negative samples tested MTB-negative (specificity 100%, 95%CI 97.2-100%), with 98.1% agreement (Kappa: 0.959). In the Xpert-Ultra survey, 70/70 (sensitivity 100%, 95%CI 94.9-100%) pools containing ≥ 1 MTB-positive sample tested MTB-positive and 140/140 (specificity 100%, 95%CI 97.4-100%) pools containing only MTB-negative samples tested MTB-negative, with 100% agreement (Kappa: 1).

In Nigeria, 46/50 (92%, 95%CI 80.8-97.8%) positive pools tested Xpert MTB/RIF MTB-positive and 71/75 (94.7%, 95%CI 86.9-98.5%) negative pools tested MTB-negative (agreement 93.6%, Kappa = 0.867). In comparison, 36/42 (86%, 95%CI 71.5-94.6%) positive pools tested Xpert-Ultra MTB-positive and 82/83 (98.8%, 95%CI 93.5-99.8%) negative pools tested negative (agreement 94.4%, Kappa = 0.871). There was no statistically significant difference in sensitivity (p-value = 0.33) or specificity (p-value = 0.14) for pooling with Xpert MTB/RIF or Xpert Ultra.

In Brazil, 99 pools were tested, of which 62 (62.6%) had MTB-detected and 37 (37.4%) MTB-not detected, including six (6.1%) with MTB-trace. The agreement of individual and pooled testing was 96.0% (Kappa of 0.913). Pooling had sensitivity of 95.3% (95%CI 86.9–99%) and specificity of 97.1% (95%CI 85.1–99.9%).

There was no significant difference in the overall agreement across all studies with individual testing when pooling either Xpert MTB/RIF (96.4% agreement (n = 323/335, CI 95% 93.7-98.1%) or Ultra (97.2% agreement (n = 422/434, CI 95% 95.1-98.5%), p-value = 0.529.

There was also no significant difference in the overall performance across all studies when pooling with either Xpert MTB/RIF or Ultra (sensitivity 93.9% (n = 123/131, CI 95% 87.9-97.1%) vs. 97.6% (n = 166/170, CI 95% 93.7-99.2%), p-value = 0.105, and specificity 98% (n = 200/204, CI 95% 94.7-99.4%) vs. 97% (n = 256/264, CI 95% 93.9-98.6%, p-value = 0.467, respectively).

**Table 2:** Agreement of individual and pooled tests

	MTB/RIF					ULTRA						
	Lao PDR		Nigeria		Overall	Lao PDR		Nigeria		Brazil		Overall
	Pooled n = 210		Pooled n = 125			Pooled n = 210		Pooled n = 125		Pooled n = 99		
**Individual**	**Neg**	**Pos**	**Neg**	**Pos**		**Neg**	**Pos**	**Neg**	**Pos**	**Neg**	**Pos**	
**All four negative**	129	0	71	4	-	140	0	82	7	34	1	-
**At least one positive**	4	77	4	46	-	0	70	1	35	3	61	-
**Agreement**	206/210 (98.1%)		117/125 (93.6%)		323/335 (96.4%)	210/210 (100%)		117/125 (93.6%)		95/99 (96.0%)		422/434 (97.2%)
**Kappa**	0.959		0.867		-	1.000		0.851		0.913		-
**Sensitivity** **(95% CI)**	95.1% (87.8–98.6%)		92.0% (80.8–97.8%)		93.9% (87.9–97.1%)	100% (94.9-100%)		85.7% (71.5–94.6%)		95.3% (86.9-99.0%)		97.6% (93.7-99.2%)
**Specificity** **(95% CI)**	100% (97.2–100%)		94.7% (86.9–98.5%)		98% (94.7–99.4%)	100% (97.4–100%)		98.8% (93.5-99.8%)		97.1% (85.1-99.9%)		97% (93.9-98.6%)

### Testing capacity and number of bacteriologically confirmed TB cases (Table 3)

In Lao PDR, pooled testing using a fixed number of 1,000 Xpert MTB/RIF cartridges would miss 5.1% (n = 10/197) of the TB cases. However, pooled testing would generate an increase of 62% in the number of people screened (1,000 vs. 1,620) leading to an increase of 54% in the absolute number of the TB cases identified despite the 10 missing TB cases (121 vs. 187 (197 − 10)). Pooled testing using a fixed number of 1,000 Ultra cartridges would generate an increase of 71.5% in the number of people tested (1,000 vs. 1,715) and 71.5% in the absolute number of TB cases identified (111 vs. 191), with no missing TB cases.

In Nigeria, pooled testing using a fixed number of 1,000 Xpert MTB/RIF cartridges would miss 5.8% (n = 13/223) of the TB cases. However, pooled testing would generate an increase of 44% in the number of people screened (1,000 vs. 1,440) leading to an increase of 45.3% in the absolute number of TB cases identified despite the 13 missing TB cases (144 vs. 210 (223 − 13)). Pooled testing using a fixed number of 1,000 Ultra cartridges would miss 9.6% (n = 27/280) of the TB cases. However, pooled testing would generate an increase of 85.8% in the number of people screened (1,000 vs. 1,858) leading to an increase of 110.7% in the absolute number of TB cases identified despite the 27 missing TB cases (120 vs. 253 (280 − 27)).

In Brazil, pooled testing using a fixed number of 1,000 Ultra cartridges would miss 3.3% (n = 9/275) of the TB cases. However, pooled testing would generate an increase of 14.2% in the number of people screened (1,000 vs. 1,142) and 10.4% in the number of TB cases identified despite the 9 missing TB cases (240 vs. 265).

**Table 3:** Cost analysis of each strategy (individual vs. pooled) by country, by assay (MTB/RIF vs. Ultra) using a fixed amount of 1,000 cartridges

Assay	Country	Testing Strategy	Number of individuals tested	Potential missed among TB cases	Bacteriologically confirmed cases
Xpert MTB/RIF	Lao PDR	Individual	N = 1,000	Reference	121
		Pooling	**N = 1,620 (62% increase)**	**5.1%, n = 10/197**	**187 (54% increase)**
	Nigeria	Individual	N = 1,000	Reference	144
		Pooling	**N = 1,440 (44% increase)**	**5.8%, n = 13/223**	**210 (45.3% increase)**
Xpert ULTRA	Lao PDR	Individual	N = 1000	Reference	111
		Pooling	**N = 1,715 (71.5% increase)**	**-**	**191 (71.5% increase)**
	Nigeria	Individual	N = 1,000	Reference	120
		Pooling	**N = 1,858 (85.8% increase)**	**9.6%, n = 27/280**	**253 (110.7% increase)**
	Brazil	Individual	N = 1,000	Reference	240
		Pooling	**N = 1,142 (14.2% increase)**	**3.3%, n = 9/275**	**265 (10.4% increase)**

### Costs of detection methods (Table 4)

#### Cost-minimization analysis

**Fig. 2:**
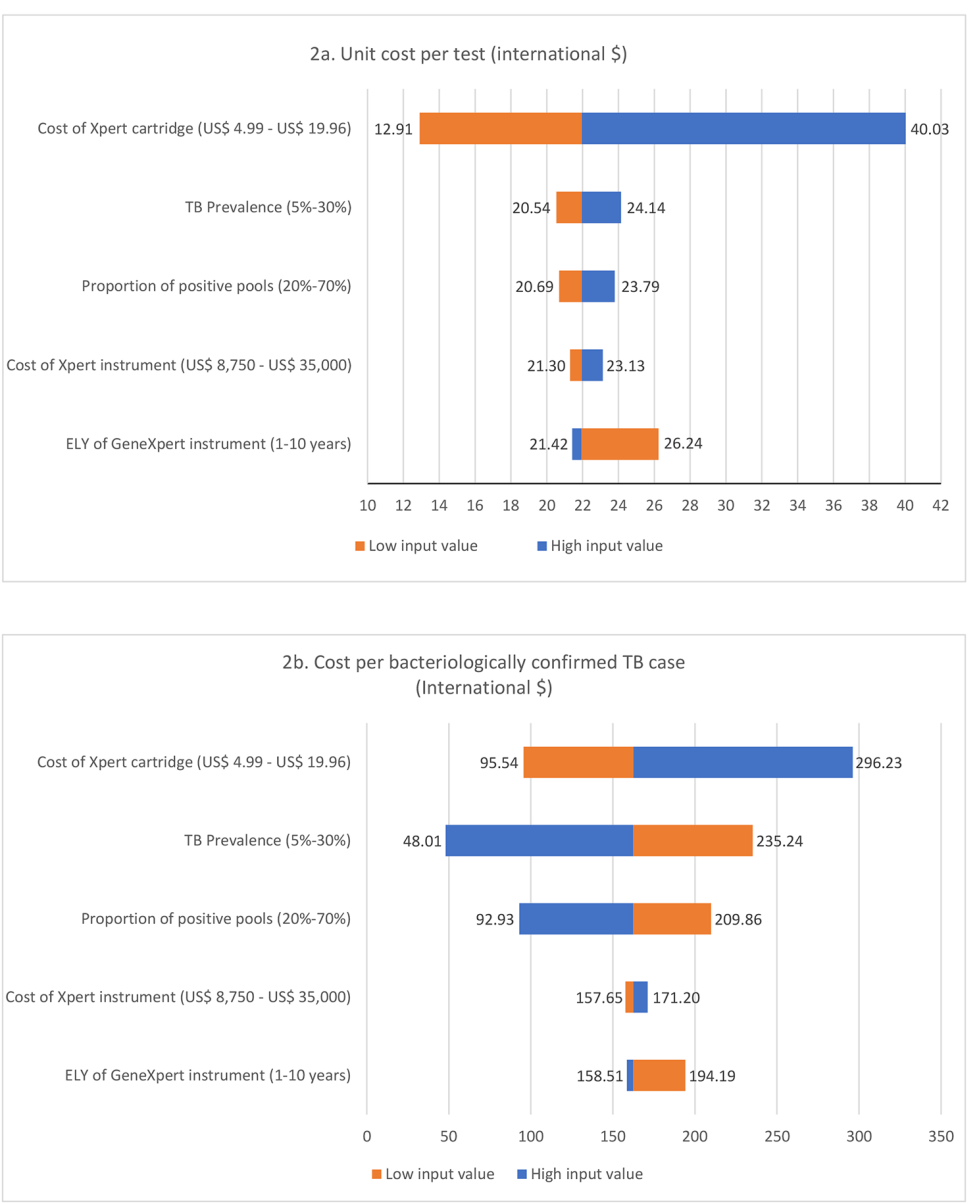
Parameters affecting the pooled testing cost-effectiveness

Since the detection of TB cases by individual and pooled testing, with both Xpert MTB/RIF and Ultra was not significantly different, we compare only the costs of tests and accept the least costly one as the cost-effective method by utilizing the cost-minimization analysis technique [13]. The univariate sensitivity analysis (Fig. 2a and b) on other parameters that could affect the cost-effectiveness and that would vary among different settings shows costs of the cartridge assay was the major determinant in the unit cost per test variation, accounting for 85.2% of the cost to test one person with presumptive TB.

The overall unit cost across all studies (1,000 individual sample size population) to test one person was 34.10 international dollars for the individual testing and 21.95 international dollars for the pooled testing, resulting in a savings of 12.15 international dollars per test performed (35.6% decrease). The overall unit cost per bacteriologically confirmed TB case was 249.64 international dollars for the individual testing and 162.44 international dollars for the pooled testing (34.9% decrease).

**Table 4:** Cost analysis of each strategy (individual vs. pooled) by country, by assay (MTB/RIF vs. Ultra) to test 1,000 presumptive TB patients

Assay	Country	Testing Strategy	Proportion positive	Savings (%)	Cost per test (International $)	Nb of cartridges	Bacteriologically confirmed cases	Cost per bacteriologically confirmed TB case (International $)
Xpert MTB/RIF	Lao PDR	Individual	12.1%	Reference	40.91	1,000	121	338.07
		Pooling	36.7%	**37.9%**	**25.42**	**617**	**115**	**221.04**
	Nigeria	Individual	14.4%	Reference	31.50	1,000	144	218.77
		Pooling	40%	**34.6%**	**20.61**	**650**	**136**	**151.53**
Xpert ULTRA	Lao PDR	Individual	11.1%	Reference	40.91	1,000	111	368.53
		Pooling	33.3%	**41.2%**	**24.04**	**583**	**111**	**216.56**
	Nigeria	Individual	12.0%	Reference	31.50	1,000	146	215.77
		Pooling	28.8%	**45.7%**	**17.10**	**538**	**136**	**125.77**
	Brazil	Individual	24%	Reference	25.69	1,000	240	107.06
		Pooling	62.6%	**12.1%**	**22.57**	**876**	**232**	**97.30**
	Overall	Individual			**34.10**			**249.64**
		Pooling			**21.95**			**162.44**

**Fig. 3:**
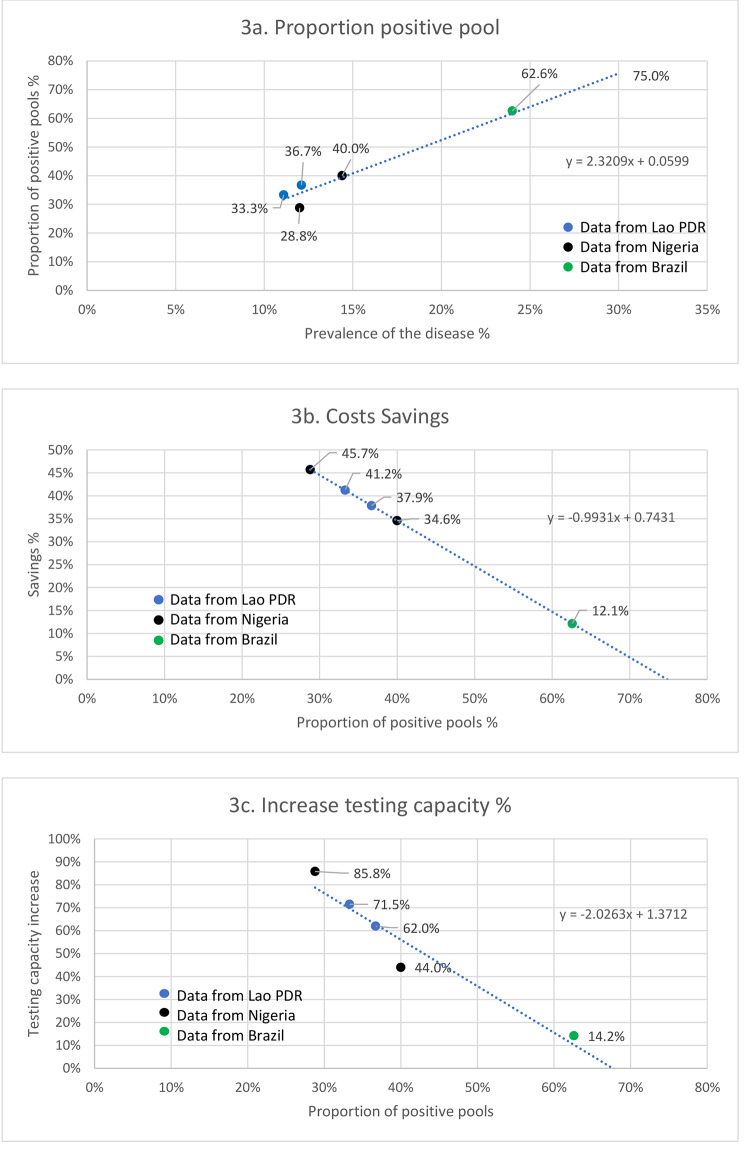
Effect of the prevalence of the disease on the amount of savings by pooling method

Figure 3a shows there is a linear correlation between the prevalence of the disease in the population tested and the proportion of positive pools. This has a direct impact on the savings: the lower the proportion of positive pools, the higher the savings in assay costs (Fig. 3b), since fewer pools require individual testing. Consequently, the lower the proportion of positive pools, the higher the increase of testing capacity (Fig. 3c).

Based on these findings, by applying a forecast forward from the trendline of the graph in Fig. 3b, we can observe savings disappear when the proportion of positive pools is ≥ 75%. Inductively, when applying a forecast forward on graph 2a, a 75% proportion of positive pools corresponds to a 30% prevalence of TB. Therefore, when the prevalence of TB is ≥ 30%, pooled testing is unlikely to still be cost-effective.

## Discussion

Our findings suggest that pooled sputum testing may improve the efficiency of GeneXpert-based testing in a variety of settings. Depending on the local TB prevalence, pooled testing could potentially enable the screening and testing of larger numbers of people more cost-effectively. Varying the number of samples per pool may also help improve cartridge savings [15]. Pooled testing demonstrated high sensitivity and specificity with both Xpert MTB/RIF and Xpert Ultra. At a time when international donors are increasingly requesting countries to commit to co-financing mechanisms for the procurement of tests from government-funded schemes, the pooling method is relevant to help National TB Programs cope with these funding gaps.

Discrepancies between individual and pooled tests only occurred among pauci-bacillary samples with high Xpert CT values. This suggests that some samples with low DNA concentrations fall below the assay’s limit of detection once mixed in the pool. Consequently, some patients with paucibacillary disease could be missed by pooling, especially if testing is based on Xpert MTB/RIF. However, if we look at the resources needed to screen this fixed number of patients, the savings will allow a higher number of patients to be tested using the same amount of resources. Therefore, under the pooling approach, a higher number of individuals could be tested leading to a higher absolute number of bacteriologically confirmed cases within a fixed time period with a fixed amount of resources, despite the number of TB cases. Pooled testing will allow a faster catch-up and more cost-effective strategy to find the people with TB compared to individual testing. Moreover, Cepheid will discontinue the production of the Xpert/MTB RIF assay in 2023, and the Global Laboratory Initiative from the Stop TB Partnership, has issued practical guidance to plan and implement a smooth transition from use of Xpert MTB/RIF to Xpert MTB/RIF Ultra cartridges, ensuring uninterrupted service and avoiding cartridge wastage [16]. If countries choose to implement pooled testing going forward, only Xpert Ultra will be available, which has better sensitivity and agreement compared to Xpert MTB/RIF.

A small number of individual samples included in MTB-positive pooled test results would return an MTB-negative result when re-tested individually. These are unexpected results since the Xpert MTB/RIF and the Ultra are highly specific and are not expected to yield false-positive results [5]. However, in other studies assessing the performance of the pooled testing for SARS-Cov-2 [17], these false-positive pooled test results happened on rare occasions with pools displaying borderline high CT values suggesting very small quantity of genetic material, and the authors have attributed it to cross-contamination during samples handling and processing. In general, for all diagnostic tests, false-positive results occur more frequently in low prevalence settings [18], and this is why for instance the WHO recommends repeat Xpert test with a fresh sample whenever rifampicin resistance is detected for an individual from groups with low risk of RR/MDR-TB, despite the high specificity of the assay [2]. It is therefore important to properly organize the workflow of samples with adequate laboratory commodities, clear standard operating procedures to avoid any clerical errors or risks of contamination. The interpretation of trace results should also be interpreted cautiously. If a pool returns a trace result, all samples included in that pool should be retested individually in order to determine if the pooled trace result is due to a very low load of bacilli that became trace due to the dilution effect, or if a trace sample was indeed included in the pool. If individual testing of samples from the trace pooled test shows there was a sample with a very low result, or one or more trace results samples, those patients need to be managed according to their national diagnostic algorithm considering pulmonary or extrapulmonary TB, HIV status, age, and prior TB treatment. However, since all samples included in MTB-positive pools are re-tested individually, the false-positive pooled test results would have no impact since the individual test result is used to guide the clinical management of the patients.

Our results demonstrate that pooling samples can significantly increase testing capacity, while simultaneously reducing the resources needed for TB mass testing. The unit cost for testing each person with presumptive TB and the savings were a function of the estimated underlying prevalence of the disease (proportion of people with MTB-positive results) in the setting where the pooled testing was implemented and their distribution within the pools. When the proportion of individuals with positive tests is lower, there are more MTB-negative pooled tests which do not require further testing, leading to higher savings. In the study from Brazil [9], the proportion of individual samples MTB-detected was much higher (24%), and many more MTB-detected pools required further individual testing (62.6%), resulting in reduced cost savings (12%). Pooling therefore works well when there is a low TB prevalence, with more negative than positive results [19]. This is an important practical factor to consider before implementation of pooled testing, as the proportion of positive pools varies significantly according to the population to be tested. Extrapolations from results reported here confirm the findings from previous study showing that in population where the disease prevalence is above 30%, the proportion of pools returning an MTB-positive results would be high (75%), leading to no savings due to the high number of deconvolution [20]. Adjusting the number of samples per pool may increase the efficiency of pooling based on the expected prevalence [15]. Pooling is not a universal solution and National TB Programmes need to be cautious as to where and when to apply it. Laboratories should determine the TB prevalence based on a rolling average of the positivity rate of their own testing and for different populations/groups. Indeed, clinical history of the patients to be tested by the pooling method must be considered, especially in settings where HIV is prevalent. PLHIV have low sputum bacillary loads, and mixing those samples into a pool with MTB-negative samples will increase the risk of getting a false negative pooled test result due to the dilution factor. However, if the proportion of TB-HIV coinfected is high, the risk of false negative results may be minimized by the increased likelihood of samples containing more than one positive specimen in the pool. Other studies have shown the dilution effect was not homogeneous, as pools with multiple positive samples often had the same or lower CT value than individual samples [17], thus indicating that the combination of multiple positive samples in a pool increases the total amount of genetic material and compensates for the dilution effect. Laboratories can then determine when the positivity rate is low enough to justify the implementation of a pooling strategy [19]. Moreover, the use of the pooling method should be a dynamic strategy following the evolution of the TB prevalence in the selected area and the positivity rate of laboratory results.

### Study limitations

Results reported here are focused on the costs minimization of the pooled testing for TB diagnosis, but parameters included for the analysis did not encompass all actual costs. For example, the costs for maintenance of the instruments were not included. These costs comprise the price of the spare parts such as the module (900$ per refurbished module, 3000$ for new module) or the annual calibration (Xpert Check calibration kit at 450$ per kit per machine), shipment, purchasing and supply management (PSM) costs and the manpower to carry out the calibration or replace faulty elements. Maintenance and servicing were recognized as major bottlenecks for the scale up of the GeneXpert instrument to a lower level in the laboratory network [6]. The absence of local authorized service providers from Cepheid and limited capacity of end-users for maintenance have led to high rates of module failures in different settings [21]. Including maintenance costs in the analysis would therefore significantly increase the actual unit cost of the test but would likely make pooling more cost-savings.

Secondly, we have not included the costs for repeating all Xpert tests with non-valid results (invalid, error, no results) in the calculation. This is an important factor because the rate of non-valid Xpert results can significantly vary from one setting to another, impacting the costs and cost-effectiveness of the pooled testing. Some studies have reported abnormally high rates of non-valid results, with 10.6% (range 5.9–16.3%) in nine countries implementing Xpert MTB/RIF [22], 7.2% (range 4–17%) in India [6], and 11% for Nigeria [23]. These high rates of non-valid results were attributed to either the environment with high temperature and/or dust, or due to poor adherence to standard operating procedures.

Thirdly, this study focuses on the cost minimisation for the diagnosis of TB using pooled testing compared to individual testing. We therefore did not assess the impacts of earlier TB diagnosis and TB treatment initiation, nor did we incorporated into the analysis the cost-effectiveness of preventing additional disease transmission. Cost-effectiveness analyses are more robust when the number of people correctly diagnosed and started on treatment is included along with costs and outcomes related to treatment, survival and disability, using cost per disability-adjusted life year (ref). Many model-based economic evaluations (ref) predicted that Xpert would be cost-effective through a reduction in tuberculosis-related mortality and/or reduction in the overtreatment of tuberculosis (ref). Given that more cases are detected with pooling, more patients will be initiated on treatment leading to less transmission, so likely that the pooling strategy would be more cost-effective if these parameters are incorporated into the model.

## Conclusions

Our results demonstrate the repeatability, reliability, consistency, and accuracy of the pooling method in a variety of settings with both Xpert MTB/RIF and Xpert Ultra, in PCF approach. The low frequency of false-negative results and the high degree of specificity makes this approach a cost-effective strategy for large scale TB testing at reduced costs. This can allow resource limited countries to catch up with the WHO End TB strategy targets despite the reversal of progress due to the Covid-19 pandemic.

## Data Availability

The datasets generated during and/or analysed during the current study are available from the corresponding author on reasonable request.

## References

[1] World Health Organization. Global tuberculosis report 2021. 2021.

[2] World Health Organization. WHO consolidated guidelines on tuberculosis. Module 3: diagnosis - rapid diagnostics for tuberculosis detection, 2021 update. 2021. 34314130

[3] Lawn SD, Nicol MP. Xpert MTB/RIF assay: development, evaluation and implementation of a new rapid molecular diagnostic for tuberculosis and rifampicin resistance. 2011(1746 – 0921 (Electronic)). 10.2217/fmb.11.84 PMC3252681 21958145

[4] Chakravorty S, Simmons AM, Rowneki M et al. The New Xpert MTB/RIF Ultra: improving detection of Mycobacterium tuberculosis and Resistance to Rifampin in an assay suitable for point-of-care testing. mBio 2017: 8(4). 10.1128/mBio.00812-17 PMC5574709 28851844

[5] World Health Organization. WHO meeting report of a technical expert consultation: non-inferiority analysis of Xpert MTF/RIF Ultra compared to Xpert MTB/RIF. 2017.

[6] Raizada N, Sachdeva KS, Sreenivas A et al. Feasibility of decentralised deployment of Xpert MTB/RIF test at lower level of health system in India. PLoS ONE 2014: 9(2): e89301. 10.1371/journal.pone.0089301 PMC3935858 24586675

[7] Cuevas LE, Santos V, Lima SVMA (2021). Systematic review of pooling Sputum as an efficient method for Xpert MTB/RIF tuberculosis testing during the COVID-19 pandemic. Emerg Infect Disease J.

[8] Iem V, Chittamany P, Suthepmany S (2022). Pooling sputum for Xpert MTB/RIF and Xpert Ultra testing during the Covid-19 pandemic in Lao People’s Democratic Republic. PLOS Global Public Health.

[9] Santos VS, Allgayer MF, Kontogianni K et al. Pooling of sputum samples to increase tuberculosis diagnostic capacity in Brazil during the COVID-19 pandemic. Int J Infect Dis 2023. 10.1016/j.ijid.2023.01.009 PMC9834119 36642209

[10] World Health Organization. Implementing the WHO Stop TB Strategy: A Handbook for National Tuberculosis Control Programmes. 2008: 1, Case detection. 26269864

[11] World Health Organization. WHO consolidated guidelines on tuberculosis. Module 2: screening – systematic screening for tuberculosis disease. 2021. 33822560

[12] Landis JR, Koch GG (1977). The measurement of observer agreement for categorical data. Biometrics.

[13] Drummond MF, Sculpher MJ, Claxton K, et al. Methods for the economic evaluation of Health Care Programmes. Oxford University Press; 2015.

[14] Foundation for Innovative New Diagnostics. GeneXpert negotiated prices. 2021.

[15] Abdalhamid B, Bilder CR, McCutchen EL (2020). Assessment of Specimen Pooling to conserve SARS CoV-2 Testing Resources. Am J Clin Pathol.

[16] Global Laboratory Initiative. Planning for country transition to Xpert MTB/RIF Ultra cartridges. 2017.

[17] Iem V, Xangsayarath P, Chittamany P (2022). Pooling samples to increase testing capacity with Xpert Xpress SARS-CoV-2 during the Covid-19 pandemic in Lao People’s Democratic Republic. PLoS ONE.

[18] Centers for Disease Control and Prevention. Guidance for Antigen Testing for SARS-CoV-2 for Healthcare Providers Testing Individuals in the Community. 2022.

[19] Centers for Disease Control and Prevention. Interim Guidance for Use of Pooling Procedures in SARS-CoV-2 Diagnostic and Screening Testing. 2021.

[20] Williams BG. Optimal pooling strategies for laboratory testing. 2010; p. arXiv:1007.4903.

[21] Albert H, Nathavitharana RR, Isaacs C (2016). Development, roll-out and impact of Xpert MTB/RIF for tuberculosis: what lessons have we learnt and how can we do better?. Eur Respir J.

[22] Creswell J, Codlin AJ, Andre E (2014). Results from early programmatic implementation of Xpert MTB/RIF testing in nine countries. BMC Infect Dis.

[23] Gidado M, Nwokoye N, Nwadike P (2018). Unsuccessful xpert MTB/RIF results: the nigerian experience. Public Health Action.

